# Yellow Jasmine

**Published:** 1861-07

**Authors:** 


### Yellow Jasmine.

In the gelseminum properties reside which are found in no other remedial agent known to pharmacologists. While we have direct arterial and cerebral sedatives in abundance, no article, in use by the physician, so completely allays local or general nervous irritability as the plant which heads this article. In it is found a truly nervous sedative, controlling not only unnatural excitement of the nervous system, but depressing the nervous or vital energies much below the normal standard. Fever being the result of irritability of the nervous system from local or general causes, is allayed, at least temporarily by it; and local disease itself, from the experiments made, seems to be under the control of this potent remedy.

In the present number of our Journal will be found an article from the pen of our friend and pupil Dr. Thomas A. Means, of Memphis, Tennessee, now in the Medical Department of the Confederate army, on the treatment of Gonorrhœa with gelseminum alone. The statistical statement of the cases treated with the remedy shows conclusively that local disease, even of specific character, is perfectly relieved by it. We invite attention to the article in our original department.

